# COVID-19 Vaccine Booster Dose Willingness among Patients with Inflammatory Bowel Disease on Infliximab and Vedolizumab: A Cross-Sectional Study

**DOI:** 10.3390/vaccines10081166

**Published:** 2022-07-22

**Authors:** Mohammad Shehab, Fatema Alrashed, Ahmad Alfadhli

**Affiliations:** 1 Department of Internal Medicine, Mubarak Al-Kabeer University Hospital, Jabriya 46300, Kuwait; ahalfadhli@moh.gov.kw; 2 Department of Pharmacy Practice, Faculty of Pharmacy, Health Sciences Center (HSC), Kuwait University, Jabriya 46300, Kuwait; fatema.alrashed@ku.edu.kw

**Keywords:** COVID-19, willingness, IBD, biologics, hesitancy, infliximab, vedolizumab

## Abstract

Background: Vaccination has been effective in preventing COVID-19 infections and related mortality. However, waning immunity after two-dose vaccination prompted health authorities to recommend a third dose of COVID-19 vaccine to boost immunity. The aim of our study was to assess willingness to receive a third (booster) dose among patients with inflammatory bowel disease (IBD). Methods: A cross-sectional study was performed at an IBD tertiary care center. Patients were recruited at the infusion room from 1 January 2022 to 31 March 2022. The primary outcome was the prevalence of a third (booster) dose of the BNT162b2 vaccine in infliximab- or vedolizumab-treated patients with IBD. The secondary outcome evaluated whether the prevalence of a third (booster) dose of the BNT162b2 vaccine differed based on type of COVID-19 vaccine, gender, age, type of biologic therapy, and citizenship. Results: In total, 499 patients with IBD were included in this study. The median age was 34.5 years, and 60% had ulcerative colitis (UC). Among the study participants, 302 (60.5%) patients were vaccinated with BNT162b2, and 197 (39.5%) were vaccinated with ChAdOx1 nCoV-19. Of the total number of participants, 400 (80.2%) were receiving infliximab, and 99 (19.8%) were receiving vedolizumab. Overall, 290 (58.1%) of the included patients were willing to receive the third (booster) dose. Patients vaccinated with BNT162b2 were more likely to be willing to receive a booster dose compared to patients vaccinated with ChAdOx1 nCoV-19 (201 (66.5%) vs. 103 (52.0%), *p* = 0.014). Infliximab-treated patients were more likely to be willing to receive a booster dose compared to patients receiving vedolizumab (310 (77.5%) vs. 62 (62.6%), *p* = 0.002). There was no statistical difference in willingness to receive a booster dose in terms of age, nationality, or gender. Conclusions: The percentage of patients with IBD willing to receive or having already received a third (booster) dose of BNT162b2 vaccine was lower compared to the general population. In addition, patients who received two doses of BNT162b2 vaccines were more likely to be willing to receive a third (booster) dose compared to patients who received ChAdOx1 nCoV-19. Patients treated with infliximab were more likely to be willing to receive a third (booster) dose of COVID-19 vaccine.

## 1. Introduction

The global spread of COVID-19 has led to multiple vaccines becoming available and approved for use. Typically, the severe acute respiratory syndrome Coronavirus 2 (SARS-CoV-2) vaccines comprise a two-dose series to generate full immunity. However, as new variants emerge, uncertainty about duration of immunity after the first two doses and possible risk of breakthrough infection led to approval of a third (booster) dose of a COVID-19 vaccine [1].

Patients with inflammatory bowel diseases (IBDs), namely Crohn’s disease (CD) and ulcerative colitis (UC), often require immune-modifying treatment, which might increase the risk of opportunistic infection [2]. Therefore, a routine check of their vaccination history is performed at diagnosis or before immunosuppressive treatment is started. Despite the increased risk of opportunistic infections with some immunosuppressive therapy, a growing body of evidence has demonstrated that IBD patients are not found to be at an increased risk of developing Coronavirus disease 2019 (COVID-19) or of experiencing a more severe disease [3,4]. In addition, studies have shown that antibody response post-COVID-19 vaccination is robust in patients with IBD treated with immunosuppressive therapy [5,6]. Therefore, vaccination is strongly encouraged in patients with IBD as the benefits of immunization outweigh the risks or the possibility of suboptimal response [7,8].

In February 2020, Kuwait reported the first confirmed case of COVID-19 in the country, and in April 2020 the first death due to COVID-19 was announced [9]. Vaccination was initiated in December 2020. BNT162b2 and ChAdOx1 nCoV-19 vaccines were the only available vaccines initially, but at later stages of the pandemic the mRNA-1273 vaccine was also approved. However, the BNT162b2 vaccine is the only approved booster vaccine in Kuwait. As of 22 May 2022, a total of 8,022,755 vaccine doses have been administered [9]. A previous study published in December 2021 explored the prevalence of COVID-19 vaccination in patients with IBD on biologic therapies in Kuwait [10]. The study found that compared to the general population, the overall prevalence of COVID-19 vaccination in patients with IBD was lower.

Evidence of the safety and efficacy of a third (booster) dose of SARS-CoV-2 vaccine is growing [8,11]. Reassuringly, the majority of currently available survey studies have reported a high willingness among patients with IBD to get vaccinated against SARS-CoV-2 [12]. However, willingness to receive a third (booster) dose in patients with IBD is still unknown. The aim of our study was to assess willingness to receive a third (booster) dose of BNT162b2 among patients with IBD in Kuwait and to evaluate possible factors associated with unwillingness to receive a third (booster) dose of COVID-19 vaccine.

## 2. Material and Methods

A cross-sectional study was performed at a tertiary care inflammatory bowel disease center, Mubarak Al-Kabeer University Hospital, in Kuwait. Patients were recruited at the gastroenterology infusion room from 1 January 2022 until 31 March 2022. Third (booster) dose SARS-CoV2 vaccination counseling was performed by health care providers during clinic and infusion room visits before recruitment. Eligibility criteria included that patients must: (1) have a confirmed diagnosis of inflammatory bowel disease before the start of the study, (2) have received two doses of SARS-CoV-2 vaccine before the start of the study, (3) have been on infliximab or vedolizumab for at least six weeks prior to recruitment, and (4) be 18 years of age or older. We excluded patients: (1) with confirmed or suspected SARS-CoV-2 infection after the second dose of vaccination, (2) who were on concurrent corticosteroids within two weeks of the start of the study, and (3) who developed severe adverse effects from a previous SARS-CoV-2 vaccination dose. Reliability was performed by evaluating the vaccination status of participants in two different visits to the infusion room, with eight weeks separating each visit. Electronic medical records of included patients were used to obtain disease characteristics, demographics, and vaccination details. The study was performed and reported in accordance with the Strengthening the Reporting of Observational Studies in Epidemiology (STROBE) guidelines (Table S1) [13].

The primary outcome was the percentage of patients with IBD on infliximab or vedolizumab who were willing to receive or had already received a third (booster) dose of BNT162b2 vaccine. The secondary outcome evaluated whether the prevalence of a third (booster) dose of the BNT162b2 vaccine differed based on type of COVID-19 vaccine, gender, age, type of biologic therapy, and citizenship.

The international classification of diseases (ICD-10 version: 2019) was used to make diagnoses of IBD. Patients were considered to have IBD when they had ICD-10 K50, K50.1, K50.8, and K50.9 indicating Crohn’s disease (CD) or ICD-10 K51, K51.0, K51.2, K51.3, K51.5, K51.8, and K51.9 indicating ulcerative colitis (UC) [14].

Descriptive analyses were conducted to calculate frequencies and proportions of categorical variables. The Chi-squared test (X^2^) was used to evaluate whether the difference in willingness to receive third (booster) dose differed across categories of demographic variables. The statistical significance level was set at α = 0.05. All analyses were conducted using R (R core team, 2017).

## 3. Results

From 1 January 2022 to 31 March 2022, 575 patients were interviewed, of which 76 patients were excluded because of confirmed SARS-CoV-2 infection after their second dose of vaccine. Therefore, 499 patients with inflammatory bowel disease (IBD) were included in this study. The median age was 34.5 years, and more than half of the included patients were female (54.9%). Out of all included patients, 40% had Crohn’s disease (CD), and 60% had ulcerative colitis (UC). The median BMI was 25.1 kg/m^2^, and 109 (22.0%) of the participants were smokers. Asthma (11.2%), diabetes (8.0%), and hypertension (6.6%) were the most reported comorbidities among the study participants. The median duration of infliximab and vedolizumab therapy at the time of the study was 12 and 11 months, respectively (Table 1).

Among the study participants, 302 (60.5%) patients were vaccinated with BNT162b2, and 197 (39.5%) were vaccinated with ChAdOx1 nCoV-19. Of the total number of participants, 400 (80.2%) were receiving infliximab, and 99 (19.8%) were receiving vedolizumab. Among infliximab-treated patients, 275 (68.7%) patients were receiving concomitant immunosuppressive therapy. Regarding age, 399 (80.0%) participants were below the age of 40, and 100 patients (20.0%) were age 40 or above. In terms of nationality, 346 (69.3%) participants were citizens of Kuwait and 153 (30.7%) were expatriates.

**Figure 1:**
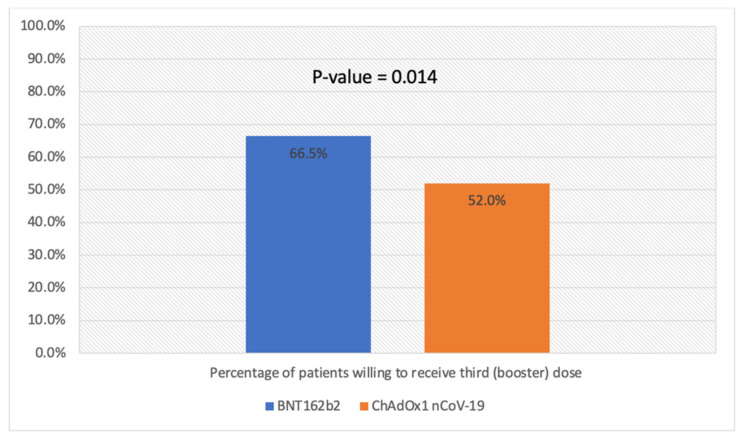
Percentage of patients willing to receive third (booster) dose stratified by type of previous vaccine.

**Figure 2:**
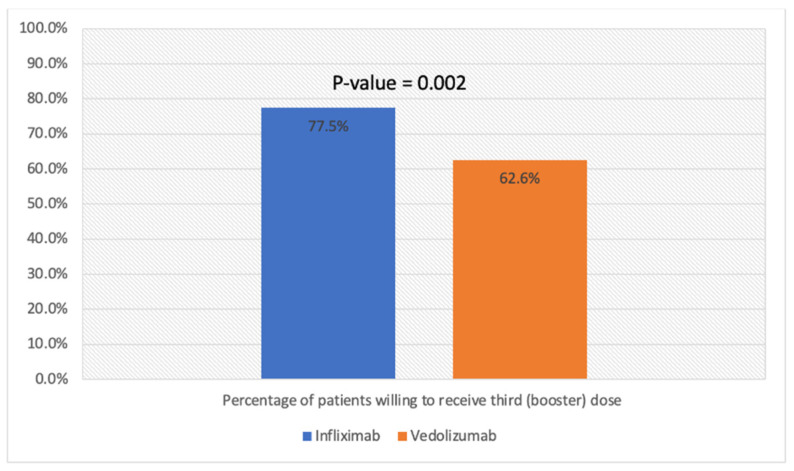
Percentage of patients willing to receive third (booster) dose stratified by biologic therapy.

Overall, 290 (58.1%) of the included patients were willing to receive the third (booster) dose. Patients with IBD vaccinated with BNT162b2 were more likely to be willing to receive the booster dose compared to patients vaccinated with ChAdOx1 nCoV-19 (201 (66.5%) vs. 103 (52.0%), *p* = 0.014) (Figure 1). In terms of biologic therapy, patients receiving infliximab were more likely to be willing to receive the booster dose compared to patients receiving vedolizumab (310 (77.5%) vs. 62 (62.6%), *p* = 0.002) (Figure 2). There was no statistical difference in willingness to receive the booster dose in terms of age, nationality, or gender (Table 2).

## 4. Discussion

In this study, 499 patients with IBD were recruited to evaluate the willingness to receive a third (booster) dose of BNT162b2 vaccine after receiving two doses of either BNT162b2 or ChAdOx1 nCoV-19 vaccines, which were the only available COVID-19 vaccines in Kuwait at the time of the study. Overall, 290 (58.1%) of the included patients received or were willing to receive the third (booster) dose. This is lower than the percentage of patients who received the third (booster) dose among the general population (77.5%) [15]. Similar to our study, a study conducted in Ontario, Canada, found that among patients with IBD, 58.3% had three doses as of 9 January 2022. Interestingly, the percentage of patients who received the third (booster) dose among patients with IBD was higher than that among the general population in Canada (44.3%) [16].

Another finding of this study was that patients who received two doses of the BNT162b2 vaccine were more likely to be willing to receive a third (booster) dose compared to patients who received the ChAdOx1 nCoV-19 vaccine initially. The reduced willingness of patients to receive a third (booster) dose after receiving the ChAdOx1 nCoV-19 vaccine could possibly be explained by reports of thromboembolic events among people who had received the ChAdOx1 nCoV-19 vaccine [17]. Uptake of vaccines is highly dependent on public trust in the safety of vaccines; therefore, concerns of safety could be a possible reason for reluctance towards vaccination [18]. Another possible reason for reluctance to receive the third (booster) dose could be experiencing more adverse events after ChAdOx1 nCoV-19 vaccination. One study found that both vaccines induced transient side effects that ranged from mild to severe; however, ChAdOx1 nCoV-19 induced more severe side effects than BNT162b2 [19]. In addition, some patients who were vaccinated with the ChAdOx1 nCoV-19 vaccine were reluctant to take a booster dose of BNT162b2 vaccine as they were concerned about the safety of mixing different type of vaccines.

In our study, there was no significant difference in the uptake of the third (booster) dose between males and females. Unlike our study, gender was found to be one of the determinants of attitude towards COVID-19 vaccination in one study conducted in China [20]. That study found that COVID-19 vaccination hesitancy existed in 50.7% of patients with IBD and merely 16.0% of participants opted for vaccination. The authors also found that the attitude of patients with IBD towards the third (booster) dose of COVID-19 vaccine was significantly more negative than that of the general population in China. Furthermore, 21.7% of patients with IBD considered the recommendations of their attending physicians as one of the three main reasons for getting vaccinated. Finally, the study demonstrated that safety, adverse events, and efficacy of COVID-19 vaccines are the top three concerns of patients with IBD for COVID-19 vaccination.

We also found that patients with IBD treated with infliximab were more likely to be willing to receive a third (booster) dose of COVID-19 vaccine compared to patients with IBD treated with vedolizumab. A possible explanation for this is that immunogenicity in patients treated with infliximab was attenuated after two doses of ChAdOx1 nCoV-19 or BNT162b2 vaccines [21,22]. This may prompt patients to be more willing to receive a third (booster) dose. A study [23] surveyed individuals with IBD to explore factors associated with vaccine uptake, concerns, and which sources of information were considered trustworthy surrounding vaccination. Authors found that self-perceived risk of being more unwell with COVID-19 due to IBD and past influenza vaccination were positive predictors of COVID-19 vaccine uptake in IBD patients. Concerns about an IBD flare with vaccination is a unique consideration in those who are vaccine hesitant and is a negative predictor of vaccine uptake.

Another study [24] assessed COVID-19 third (booster) dose willingness and hesitancy in Italian IBD patients and investigated the determinants of these attitudes. Authors reported that more than four in five (80.3%) IBD patients were ready to receive the third (booster) dose. Adherence to previous vaccinations had a positive influence on willingness to receive the third (booster) dose. The study also observed positive association between vaccine willingness and a perceived higher risk of COVID-19 because of IBD. Finally, authors found that the most common reasons for COVID-19 vaccine hesitancy were the fear of adverse events and concerns about a perceived too-fast development.

Factors that increase the likelihood of receiving the third (booster) dose of COVID-19 vaccines in the general population have been explored extensively in the literature. One study [25] measured factors associated with COVID-19 vaccine booster hesitancy and assessed the roles of vaccine literacy and vaccine confidence in American adults. A total of 2138 Americans aged 18 and over participated in this study. Among participants who were fully vaccinated or who had received the first dose, an overwhelming majority (79.1%) would take a booster dose if recommended. In contrast, nearly half of the unvaccinated participants (46.3%) reported they would not take the booster dose. The study cited different factors that influenced this decision including socioeconomic factors, education level, and demographic factors. In particular, those with a college education and graduate degrees were significantly more likely to accept third (booster) doses than those with only a high school education.

Furthermore, a questionnaire-based study was conducted in Algeria to determine COVID-19 vaccine booster acceptance and its associated factors in the general population [26]. Authors found that the most common reasons for acceptance were experts’ recommendations (24.6%) and the belief that COVID-19 vaccine boosters were necessary and efficient, while rejection was mainly due to the belief that primer doses are sufficient (15.5%) or that vaccination in general is inefficient (8%). Males, older individuals, those with chronic comorbidities or a history of COVID-19 infection, non-healthcare workers, and those with low educational levels were associated with significantly higher odds for booster acceptance.

Different international gastroenterological societies have published their recommendations regarding a third (booster) dose of COVID-19 vaccination. The International Organization for the Study of Inflammatory Bowel Disease [8] (IOIBD) recommends that all patients with IBD receive any of the available COVID-19 vaccines, as bad outcomes have been documented in patients with active IBD infected with SARS-CoV-2. The British Society of Gastroenterology [7] (BSG) also recommends early vaccination for patients with IBD, as the benefits of vaccination outweigh the risks. Furthermore, the BSG recommends that immunomodulators should not be withheld for vaccination, and conversely, that vaccination should not be deferred due to IBD medications. The Crohn’s & Colitis Foundation [27] (CCF) largely agreed with the IOIBD. Additionally, the CCF was supportive of patients receiving additional doses if they were on immunosuppressive therapies. The CCF followed guidelines from the US Centers for Disease Control and Prevention stating that most patients with IBD, aside from patients on no medications or mesalamines only, qualified for an early booster [28]. It is essential that gastroenterologists are appropriately updated on efficacy and safety of the available COVID-19 vaccines as they play an important role in patients’ education as well as encouraging patients with IBD to get vaccinated. Furthermore, the effectiveness of the fourth dose of BNT162b2 is currently being investigated and new data are emerging regarding the best usage of the fourth dose [29].

High levels of B-cell immunity might be necessary to counterbalance the curtailment of cellular immunity effectiveness, and can be achieved by receiving a booster dose of COVID-19 vaccine. However, interestingly, there is some evidence that patients on anti-TNF therapy are less likely to experience severe COVID-19. Because patients with IBD are already anxious about getting sick with COVID-19, it should be made clear to them that although their cellular immunity may be reduced with anti-TNF therapy, they are not at increased risk of experiencing worse COVID-19 related outcomes compared to the general population [30].

The results of this study can be used to help physicians (especially IBD specialists), researchers, and public health departments to understand factors behind unwillingness to receive third (booster) doses. This, in turn, can be used to support the creation of measures and interventions to encourage patients with IBD to receive a third (booster) dose. However, there are limitations to our study. Given that this is a cross-sectional observational study, selection bias and confounding variables are significant challenges. As a result of the complexity of biases that can affect an observational study, it is virtually impossible to conclude that our results are not affected, in totality or in part, by different variables that cannot be controlled for. Examples of variables that could have affected the results are time and booster dose priority. At the initial phase of vaccination, the third (booster) dose was available only to certain groups such as the elderly. In addition, given the rapidly evolving nature of the pandemic, patients’ willingness to receive a booster dose may change over time. Therefore, readers must carefully assess for possible explanations and associations. Finally, patients with IBD on other biologic therapies such as adalimumab and ustekinumab were not evaluated.

## 5. Conclusions

The percentage of patients with IBD who are willing to receive or have already received a third (booster) dose of COVID-19 vaccine was lower compared to general population. In addition, patients who received two doses of the BNT162b2 vaccine were more likely to be willing to receive a third (booster) dose compared to patients who received the ChAdOx1 nCoV-19 vaccine. Patients treated with infliximab were more likely to be willing to receive a third (booster) dose of COVID-19 vaccine. Health education and recommendation from authoritative sources, such as government and IBD specialists, may support increased uptake of the third (booster) dose in patients with IBD.

## Figures and Tables

**Table 1:** Demographics of patients with IBD.

Variable	Study Group (*n* = 499)
Mean age (years)	34.5
Sex *n* (%)	
Male	225 (45.1%)
Female	274 (54.9%)
BMI (Median) kg/m^2^	25.1
Smoking *n* (%)	109 (22.0%)
Comorbidities *n* (%)	
Diabetes	40 (8.0%)
Osteoarthritis	21 (4.2%)
Hypertension	33 (6.6%)
Cardiovascular Disease	11 (2.2%)
Rheumatoid Arthritis	29 (5.8%)
Asthma	56 (11.2%)
Dyslipidemia	15 (3.0%)
Median infliximab therapy (months)	12
Median vedolizumab therapy (months)	11
Disease extent, *n* (%)	
Ulcerative colitis (UC)	200 (40.0%)
E1: ulcerative proctitis	36 (17.8%)
E2: left sided colitis	57 (28.5%)
E3: extensive colitis	107 (53.6%)
Crohn’s disease (CD)	299 (60.0%)
L1: ileal	149 (50.0%)
L2: colonic	35 (11.9%)
L3: ileocolonic	105 (35.2%)
L4: upper gastrointestinal	8 (2.8%)
B1: inflammatory	133 (44.6%)
B2: stricturing	78 (26.2%)
B3: penetrating	87 (29.2%)

**Table 2:** Demographics of patients with IBD according to willingness to receive the booster dose.

Demographics	Total Number of Patients (499)	Willingness		*p*-Value
		Yes 290 (58.1%)	No 209 (41.9%)	
BNT162b2	302	201 (66.5%)	101 (33.5%)	0.014
ChAdOx1 nCoV-19	197	103 (52.0%)	94 (48.0%)	
Infliximab	400	310 (77.5%)	90 (22.5%)	0.002
Vedolizumab	99	62 (62.6%)	37 (37.8%)	
Age > 40	100	59 (59.0%)	41 (41.0%)	0.292
Age < 40	399	258 (64.6%)	141 (35.4%)	
Citizens	346	250 (72.2%)	96 (27.8%)	0.206
Expatriates	153	102 (66.6%)	51 (33.4%)	
Male	225	141 (62.6%)	84 (37.4%)	0.061
Female	274	149 (54.3%)	125 (45.7%)	

## Data Availability

The data presented in this study are available on request from the corresponding author. The data are not publicly available due to legal and ethical restrictions.

## Supplementary Materials

The following supporting information can be downloaded at: https://www.mdpi.com/article/10.3390/vaccines10081166/s1, Table S1: STROBE guidelines.
